# The role of executive functions, social cognition and intelligence in predicting social adaptation of vulnerable populations

**DOI:** 10.1038/s41598-022-21985-9

**Published:** 2022-11-04

**Authors:** M. Schulte, N. Trujillo, O. A. Rodríguez-Villagra, N. Salas, A. Ibañez, N. Carriedo, D. Huepe

**Affiliations:** 1 Cognitive Neuroscience Center (CNC), Universidad de San Andrés, Buenos Aires, Argentina; 2 Mental Health Group, National Department of Public Health, University of Antioquia, UDEA, calle 62#52-59, 050010 Medellín, Colombia; 3 Neuroscience Group, Universidad de Antioquia-UDEA, 050010 Medellín, Colombia; 4 Institute for Psychological Research, University of Costa Rica, Sabanilla, San José, Costa Rica; 5 Neuroscience Research Center, University of Costa Rica, San Pedro, San José, Costa Rica; 6 Universidad Finis Terrae, Santiago de Chile, Chile; 7 Center for Social and Cognitive Neuroscience (CSCN), School of Psychology, Universidad Adolfo Ibáñez, Santiago de Chile, Chile; 8 Latin American Brain Health Institute (BrainLat), Universidad Adolfo Ibáñez, Santiago de Chile, Chile; 9 Consejo Nacional de Investigaciones Científicas Y Técnicas (CONICET), Buenos Aires, Argentina; 10 Global Brain Health Institute (GBHI) UCSF, San Francisco, USA; 11 Trinity College Dublin (TCD), Dublin, Ireland; 12 National Distance Education University (UNED), Madrid, Spain

**Keywords:** Human behaviour, Social behaviour

## Abstract

This study sought to evaluate the roles of and interactions between cognitive processes that have been shown to exhibit impact from socioeconomic status (SES) and living conditions in predicting social adaptation (SA) in a population of adults living in socially vulnerable conditions. Participants included 226 people between the ages of 18 and 60 who have been living in vulnerable contexts throughout life in Santiago, Chile. Data was collected through a battery of psychological assessments. A structural equation model (SEM) was implemented to examine the interrelationships among cognitive and social variables. Results indicate a significant relationship between executive function (EF) and SA through both social cognition (SC) and intelligence. Theory of Mind (ToM), a component of SC, was shown to exhibit a significant relationship with affective empathy; interestingly, this was negatively related to SA. Moreover, fluid intelligence (FI) was found to exhibit a positive, indirect relationship with SA through crystallized intelligence (CI). Evaluation of these results in the context of research on the impacts of SES and vulnerable living conditions on psychological function may allow for the development of more effective clinical, political, and social interventions to support psychosocial health among socially vulnerable populations.

## Introduction

Social adaptation (SA) encompasses an ability to interact with others in a manner that aligns with sociocultural norms^1^. Socially vulnerable populations represent individuals living in contexts with reduced access to economic resources due to their low-income range. This population lives in social risk neighborhoods and lacks the social resources necessary to withstand the impacts of external stressors^2–5^; this thwarts SA. The number and magnitude of stressors experienced by these persons combined with their insufficient access to resources to manage stress create conditions for poor mental health and chronic stress^6,7^. Furthermore, living in vulnerable conditions, particularly as a child, may be a driver for a reduction in the performance of functions or processes associated with the prefrontal cortex—that have been independently associated with SA—such as social cognition (SC) (includes decision-making, emotional processing, Theory of Mind (ToM), and empathy), fluid intelligence (FI), crystallized intelligence (CI), and executive functions (EF)^7–12^. Given the behavioral control exerted by these cognitive functions, it has been suggested that adolescents living in poverty who exhibit deficits in these functions may partake in resultant maladaptive behaviors that thwart SA^13–20^. The dynamics, precedents, and interactions of these cognitive functions in the context of SA and social vulnerability, however, are not yet fully understood. By elucidating the ways in which these cognitive constructs work together and separately in predicting social outcomes, we might better understand the role of specific variables in predicting SA. This information may allow for the design of more effective intervention programs to support psychosocial health among vulnerable populations.

Existing studies have investigated EF in relation to ToM (a component of SC) and FI against the context of SA to build models that more accurately reflect the synergies and determinants of these processes. In addition to the role of EF impairments in yielding maladaptive behavior^15,16,18–20^, these impairments are also related to reduced functioning in ToM^21,22^. In the sample investigated by Colvert et al.^21^, subjects exhibiting cognitive dysfunction related to EF and ToM were significantly more likely to have been subjected to deprived environmental conditions earlier in life^21^. This provides further indication of the role of socioeconomic status (SES) and early living conditions in determining cognitive capacities that affect SA.

Similar to EF, intelligence develops in childhood and exhibits environmental effects related to SES and early living conditions^23,24^. Poverty in childhood is associated with reduced literacy levels that exhibit a negative impact on FI^11^. Additionally, concerns related to poverty consume mental resources, leaving fewer to allocate toward other tasks^17^. Brydges et al.^25^ found a single factor model of EF—whose capacity seems to be similarly influenced by SES—that robustly predicted FI and CI capacity^25^. Though FI has not been shown to determine the expression of social cognitive abilities (including ToM), there exists evidence to suggest that this expression may be mediated by CI^26^. Given this background, intelligence seems to relate to SA both directly and indirectly. Directly, FI seems to support analysis and adaptation to changing social situations. Indirectly, CI seems to aid in perspective-taking abilities, experience, and expression which support SA.

Empathy, a component of SC, must also be considered in this context. EF has been shown to exhibit regulatory control over empathy while also serving as its developmental foundation^27–29^. While EF ability is positively correlated with empathic capacity, this relationship is specifically stronger with cognitive empathy (i.e., understanding what another is feeling) than affective empathy (i.e., feeling what another is feeling)^30–34^. Cognitive empathy recruits inhibitory control, working memory, and cognitive flexibility while affective empathy only recruits inhibitory control^34^. Despite its siloed and less significant relationship with EF, affective empathy seems to list cognitive empathy as a prerequisite to its function^31^. In keeping with its putative position as a higher-level outcome of its precedents in cognitive empathy, affective empathy has been shown to be directly related to the selection of adaptive social strategies among poor individuals. This has not been shown to be the case for wealthy individuals^35^. Given this context, it is of critical importance to consider EF and empathy in evaluating SA in individuals of low SES.

**Figure 1:**
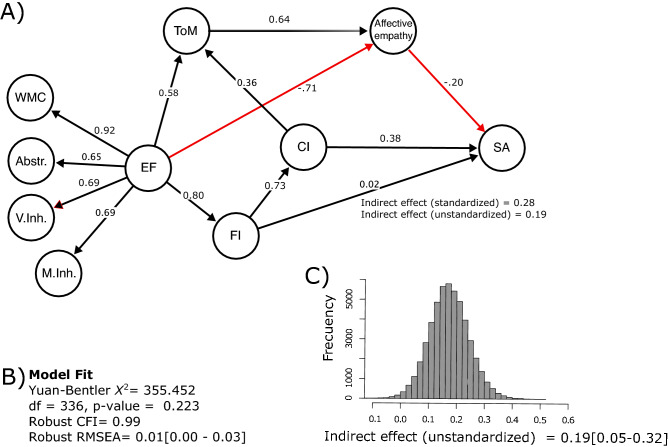
Structural equation modelling social adaptation of vulnerable populations. Main results. (**A**) Structural equation model 1; (**B**) Model fit, values in brackets denote 90% confidence intervals; (**C**) Monte Carlo simulations of the distribution of the unstandardized indirect effect of fluid intelligence (FI) on social adaptation (SA) through crystallized intelligence (CI), values in brackets denote 95% confidence intervals. Variables in circles are unobserved (latent) factors explaining observed (manifest) variables (not shown). Arrows indicate the hypothesized pathways with numbers as the standardized regression estimates. Dashed lines indicate that the effects were not statistically significant. *WMC* working memory capacity, *Abstr.* Abstraction, *V.Inh* verbal inhibitory control, *M.Inh* motor inhibitory control, *EF* executive function, *ToM* theory of mind, *FI* fluid intelligence, *AE* affective empathy, *SA* social adaptation, *M* marginal effect.

Prior studies have investigated interactions between the aforementioned cognitive functions (e.g., EF, FI, SC) in isolation and in populations temporarily experiencing low SES. However, to our knowledge, no previous studies have investigated the relationships between these constructs and the predicting roles played by these functions to better understand SA in populations longitudinally impacted by low SES. Based on the literature overview presented previously, we proposed a model in which the relationships between EF, SC, and intelligence are tested simultaneously to investigate their role in predicting SA (see Fig. 1). We expect that EF serves as a cornerstone variable that predicts, on the one hand, aspects of intelligence, and on the other, components of SC. Both factors—intelligence and SC—might contribute to the effect of EF on SA. Based on this structural model and the reviewed literature, we evaluated the following additional hypotheses: (i) a higher-order factor reflecting the common variance of EF subdomains (i.e., verbal inhibitory control, motor inhibitory control, abstraction, and working memory capacity) is directly related to latent variables reflecting ToM, FI, and affective empathy; (ii) ToM is directly related to affective empathy and the latter is directly related to SA [we did not hypothesize a direct path between ToM and FI because there is limited evidence for adults in Latin America living in long-term vulnerable contexts—however, we contrast this model with an alternative one including this path in the Supplementary Materials]; (iii) FI is directly related to CI and the latter is directly related to SA (i.e., direct effect); (iv) FI is indirectly related to SA through CI (i.e., indirect effect). Taken together, this paper aims to determine the potential of the above-mentioned cognitive features in predicting SA in adults living in socially vulnerable contexts since childhood.

## Methods

The data presented in this paper were part of a multidimensional study in which social adaptation in vulnerable populations was studied in different waves. The data presented here corresponded to the 2014–15 wave and they have not been published before.

### Participants

A total of 226 adults (51.3% female) between ages 18 to 60 took part in this study (M = 42.00, SD = 14.80). The mean years of education were around nine (M = 9.44, SD = 3.24, Range = 0–18). This equates to incomplete secondary studies. All participants have lived most of their lives in vulnerable socioeconomic contexts according to the Social Protection Sheet of the Ministry of Social Development and Family of the Chilean government^36^. All participants were qualified as a part of the 40th percentile of the lowest income range (stretch 1 of 7) of the Chilean Welfare Program^36^. This socioeconomic qualification depends on: (i) the sum of labor, pension, and capital income of all members of the household; (ii) the number of household members; (iii) characteristics of the household members: age, disability, or dependency; (iv) evaluation of goods and services to which a household has access or owns and which allow for inference of its socioeconomic status when compared to the actual income received by the household. Furthermore, most of the participants lived in neighborhoods in Santiago, Chile that have a high social risk index and participated in a social program of the Ministry of Social Development and Family.

According to the above-mentioned criteria, before starting the study, we performed a brief semi-structured interview to establish the degree of each individual’s exposure to a long-term socially vulnerable context. Only participants who met this criterion participated in the study. Participation was voluntary and data were anonymized. We excluded individuals with a visual or hearing impairment who told us they would be unable to complete our assessment battery (e.g., to read or respond to verbal information or to follow the evaluator’s oral instruction). Persons with psychiatric or neurological conditions did not participate. All participants provided a signature as proof of their informed consent. The study received prior approval from the ethics committee of the Adolfo Ibañez University (Santiago, Chile) and followed the protocol of the Declaration of Helsinki.

### Procedure

All neuropsychological tests, socio-demographic and psychological questionnaires used in this study were administered by licensed clinical social workers and clinical psychologists. Each evaluator made sure that each participant understood the informed consent before administering the tests. Participants were informed that they retained the right to clarify any confusion at any time by asking the examiner directly. The battery of assessments was randomly sequenced for each participant to reduce order bias. Participants took approximately 90 min to complete the protocol, including a 15-min break. Most of the interviews were conducted in the neighborhoods in which the participants lived—typically, in the community meeting house. The field study was carried out between 2014 and 2015.

### Instruments

The study protocol included the following measures. Estimates of reliability were calculated with the split-half methods (Spearman-Brown coefficient) for dichotomic scales and Cronbach’s *α* from Likert scales.

- (a) EF measurement with INECO Frontal Screening (IFS)^37,38^. This tool evaluates EF through a diversity of domains: motor programming, conflicting instructions, Go-No Go testing (to measure motor inhibition; M.Inh.), and verbal inhibitory control testing (to measure verbal inhibitory control: V.Inh.); proverb interpretation (to measure abstraction: Abstr.); and backwards digit span, phonological loop, and visuospatial sketchpad testing (to measure working memory capacity: WMC). IFS is a sensitive instrument and has been tested in patients with frontal lobe injuries and neuropsychiatric disorders against healthy controls^38,39^. It has been used in current populations^37^. The reliability of the Chilean version of IFS was *α* = 0.905^37^. The reliability of the sample was *α* = 0.76.
- (b) FI and CI measurement through the Wechsler Adult Intelligence Scale III (WAIS-III)^40^ and two subtests: progressive matrices, using raw (not standardized) scores (to measure FI; consists of 26 trials, so 26 is the maximum direct score) and vocabulary (to measure CI; consists of 33 words for which meaning must be explained). WAIS-III has been validated in its assessment of both SC-specific factors^41^ as well as intelligence in healthy controls and patients with frontal lobe lesions and neuropsychiatric disorders^42^. Note that we applied this version of the assessment and not WAIS-IV because their norms were published while data of the present investigation were being collected. Estimates of reliability ranged from *α* = 0.86 to 0.95 across different ages for vocabulary subscale, and *α* = 0.76 to 0.94 across ages for matrices subscale^43^. In the present study, both subscales showed high reliability for this sample—*α* = 0.79 and *α* = 0.75, respectively.
- (c) ToM measurement through the Reading the Mind in the Eyes Test and Mini-Sea. The Reading the Mind in the Eyes Test evaluates ToM through the presentation of 17 pictures of human faces^44^ in which only the area around the eyes is visible^45,46^; emotional state must be interpreted by the participant based on the image. It has been validated for its use in evaluating ToM in healthy populations^47^ as well as those who have been shown to exhibit deficits in SC (e.g., people living with schizophrenia, autism, anorexia)^45,48,49^. In complement, emotional recognition was tested with the Mini-Sea^50^. This instrument includes two subtests: (i) a facial emotion recognition test (from Ekman pictures; scored from 0 to 15) in which participants are instructed to categorize the emotion that is being expressed; and (ii) a shortened version of the Faux Pas recognition test^51^ used to evaluate ToM based on stories that describe everyday social situations. The reliability of Reading the Mind Test test was 0.56^44^. In the present study, *α* = 0.51 was obtained for the Reading the Mind Eyes and *α* = 0.78 for the Mini Sea. Although the reliability of the Reading the Mind in the Eyes Test was not good, it must be considered that calculating Cronbach’s alfa would not be suitable for this test —as is the case with other similar tests designed to measure emotion recognition, such as MiniPONS^52^. In these cases, only correct responses between individuals can be compared and thus, calculating this parameter is not straightforward^53^. However, recent studies have reported acceptable test–retest reliability for the adult version of the test in very different samples, which contributes to solving this gap^47,53–55^. Test–retest reliability for the Spanish version was 0.63 based on the interclass correlation^53^].
- (d) Affective empathy measurement through the Empathy Quotient—a 60-item questionnaire in which 40 questions explore empathy and 20 serve as fillers to avoid an excessive and conspicuous focus on empathy that might trigger biased response^56^. The affective dimension was calculated through a parcel analysis of affective items of empathy^57^. The use of the Empathy Quotient has been validated in healthy populations as well as those with depersonalization symptoms and autism spectrum disorders^58–60^. Item reliability for the EQ was 0.99^59^. In this sample, *α* = 0.75 was obtained.
- (e) SA measurement using the Social Adaptation Self-evaluation Scale (SASS). This instrument was developed as a quick and straightforward evaluation that specifically targets the measurement of social motivation and behavior of the examinee^61,62^. It contains 21 questions that explore job interest, homework interest, work enjoyment, interest in hobbies, quality of spare time, relationship-seeking behavior (familiar, romantic, and platonic), relationship quality and appreciation, sociability, social attractiveness, social compliance, community involvement, intellectual interest, communication difficulties, rejection sensitivity, vainness, difficulty in coping with resources, and control of surroundings. The use of SASS in evaluating SA has been validated in both healthy populations as well as in patients with Major Depressive Disorder and Bipolar Disorder^63^. The reliability is 0.78^61,62^. In this study, *α* = 0.90 was obtained.

### Statistical analysis

Structural Equation Modeling (SEM) analysis was used to estimate the proposed model, provide a detailed accounting of measurement errors, and accurately estimate the structural relations between latent factors^64^. Data preparation, analyses, and plotting were conducted with R in RStudio^65^ using the following packages: tidyverse^66^, lavaan^67,68^ semTools^69^ and semPlot^70^.

Since the data were not normally distributed, we used maximum likelihood estimation with robust (Huber-White) standard errors; a chi-square (χ^2^) statistic was scaled by the Yuan-Bentler correction factor^71^. A full information maximum likelihood estimation method was implemented to account for missing data^72^. For identification and scaling of the model, the variance of each latent variable was fixed to one. Structural equation modelling adequacy fit indices were used to assess model goodness of fit: the YB χ^2^ statistic, the robust comparative fit index (CFI)^73^, and the root mean square error approximation (RMSEA)^73^. The YB χ2 statistic is used as a fit index and it is expected to be as close to zero as possible, thus it is not expected to be significant (i.e., p-value should be > 0.05). The CFI is an index with values from 0 to 1 assessing the extent to which the specified model improves fit over the null model (values > 0.90 considered as adequate fit, values in the range of 0.95–0.99 considered as excellent fit, and a value of one considered as exact fit)^74^. The RMSEA indicates the discrepancies between the sample variance-covariance matrix and the model-implied variance-covariance matrix (values > 0.08 considered as poor fit, values in the range of 0.05–0.08 considered as adequate fit, and values ≤ 0.05 considered as good fit)^64^. Finally, Monte Carlo simulations were used to construct confidence intervals for the indirect effects^75^.

## Results

The fit of the model to data was excellent (see Panel B of Fig. 1) [Supplementary material presents a comparison of this model with two alternative models. Data analysis indicates that this model offers the best approximation to the data]. The structural model and standardized model parameters are shown in Fig. 1 (factor loadings are shown in Table 1). The descriptive statistics of the model variables are shown in Table 2. The EF factor was specified as a second-order factor in which seemingly distinct but related factors (i.e., WMC, Abstr.: V.Inh., and M.Inh.) are accounted for by one common underlying higher-order factor^76,77^. The factor loadings for all first-order factors (i.e., WMC, Abstr. V.Inh, and M.Inh) were statistically significant (*p* < 0.01, see Table 1). As expected, the influence of EF on ToM and FI was statistically significant (*p* < 0.01; hypothesis 1). However, EF exhibited a strong negative correlation to affective empathy (β = − 0.71, *p* = 0.094; hypothesis 1, see Fig. 1). Concerning the second hypothesis, ToM was moderately related to affective empathy (β = 0.64, *p* = 0.051). Contrary to our prediction, affective empathy was negatively related to SA (*p* < 0.05). Additionally, FI was strongly related to CI (*p* < 0.001) and the latter was related to SA (p < 0.01; hypothesis 3). In this model we also tested the hypothesis that the prediction of FI to SA may be accounted for by CI. To this aim, we estimate the direct and indirect effects of FI on SA. The direct effect is the influence of FI on SA controlling for CI. The indirect effect is the influence of FI on SA through CI. As panel A of Fig. 1 displays, the direct effect was weak and nonsignificant (direct effect β = 0.02, *p* = 0.867, see the value of the dashed black line in Fig. 1). The standardized indirect effect was small (β = 0.28) and reliable. Monte Carlo simulations for constructing confidence intervals of the unstandardized indirect effect revealed that they did not include zero (see panel C of Fig. 1). This finding suggests that the effect of FI on SA is accounted for by CI (hypothesis 4). That is, persons with better FI exhibited higher scores in CI, which in turn predicted higher levels of SA.

**Table 1:** Factor loadings for the observed variables in the SEM model.

Latent variables	Observed variables	Factor loading*
Working memory capacity (WMC)	Spatial working memory (IFS)	0.599
	Backward digits span (IFS)	0.711
	Verbal working memory (IFS)	0.377
Abstraction (Abstr.)	Proverb 1 (IFS)	0.469
	Proverb 2 (IFS)	0.532
	Proverb 3 (IFS)	0.279
Verbal inhibitory control (V.Inh.)	Sentence 1(IFS)	0.560
	Sentence 2 (IFS)	0.607
	Sentence 3 (IFS)	0.749
Motor inhibitory control (M.Inh.)	Motor programming (IFS)	0.727
	Conflict instructions (IFS)	0.668
	Go-No go (IFS)	0.668
Theory of mind (ToM)	Parcel 1 (Mini-Sea)	0.601**
	Parcel 2 (Mini-Sea)	0.639
	Parcel 1 (RME)	0.619
	Parcel 2 (RME)	0.659
Fluid intelligence (FI)	Parcel 1 (WAIS matrix reasoning)	0.771
	Parcel 2 (WAIS-III matrix reasoning)	0.771
	Parcel 3 (WAIS-III matrix reasoning)	0.710
Crystallized intelligence (CI)	Parcel 1 (WAIS-III vocabulary)	0.733
	Parcel 2 (WAIS-III vocabulary)	0.813
	Parcel 3 (WAIS-III vocabulary)	0.876
Affective empathy	Parcel 1 (EQ scale)	0.651
	Parcel 2 (EQ scale)	0.369
	Parcel 3 (EQ scale)	0.504
Social adaptation (SA)	Parcel 1 (SASS)	0.815
	Parcel 2 (SASS)	0.750
	Parcel 3 (SASS)	0.681

Parcels were constructed for Mini-Sea, RME, WAIS-III matrix reasoning, EQ scale and SASS; we followed one strategy suggested by^78^.

Specifically, parcels were constructed for reducing unwanted correlations between residual variances; that is, items with correlated residual variances were allocated to the same parcel.

This strategy of parceling can create indicators with better measurement properties^78^.

*IFS* INECO frontal screening test, *RME* reading the mind in the eyes test, *WAIS-III* wechsler adult intelligence scale III,*EQ scale* empathy quotient, *SASS* Social Adaptation Self-evaluation Scale.

All factor loadings were statistically significant (p < 0.05).

*Factor loadings between 0.0– ± 0.20, ± 0.21– ± 0.40, ± 0.41– ± 0.60, ± 0.61–0.99, and 1.0 were considered weak, low, moderate, strong, and perfect, respectively^79^.

**The model included a covariance parameter between the residual variances of Parcels 1 and 2 of Mini Sea.

**Table 2:** Descriptive statistics of the model variables.

Observed variables	Mean	SD	Median	Range	Skew	Kurtosis
Working memory capacity (WMC)	3.76	0.93	3.67	6.00	0.07	0.59
Motor inhibitory control (M.Ihn)	2.55	0.64	2.67	3.00	-1.83	3.28
Abstraction (Abstr.)	0.32	0.31	0.33	1.00	0.65	-0.50
Verbal inhibitory control (V.Inh.)	1.22	0.54	1.33	2.00	-0.68	0.06
Theory of mind (ToM)	0.64	0.12	0.65	0.64	-0.50	0.06
Affective empathy	21.59	2.89	22.58	15.08	-1.07	0.98
Fluid intelligence (FI)	0.44	0.17	0.42	0.80	0.52	-0.48
Crystallized intelligence (CI)	0.74	0.29	0.72	1.68	0.47	-0.06
INECO total (sum)	19.89	4.37	20.00	26.00	-0.65	0.78
Social adaptation (sum)	40.14	7.97	41.00	43.00	-0.52	0.20

With exception of INECO total and Social adaptation, the remaining observed/measured variables were calculated as mean across items.

Finally, in accordance with the reviewer’s request, we have conducted an additional analysis to test the SEM model proposed (Model 1) against rival models. We presented two alternative models in the Supplementary Materials section, including an additional path from FI to ToM (Model 2), and an additional path from EF to ToM (Model 3). The fit of models 1 and 2 were identical, but the path from FI to ToM was non-significant, suggesting that it is unnecessary to include it in the model. This could be indicating that, in the presence of an effect of EF to ToM, the influence of FI on ToM was not important. Thus, Model 1 was preferable. The comparison between models 1 and 3 showed similar goodness-of-fit and similar parameter estimates, but Model 1 presents the smallest AIC and ΔAIC indicates no support for Model 3. Taken together, the model proposed in this paper (Model 1) was the best representation of the data.

## Discussion

This study was conducted to evaluate the dynamics of specific cognitive features—EF, SC, and intelligence—in predicting SA among a population that has sustained low SES over their lifetime. Our main hypothesis was that EF serves as a cornerstone variable that predicts components of SC and aspects of intelligence and that both factors—SC and intelligence—might contribute to the effect of EF on SA. In evaluating our SEM according to our first hypothesis, EF was found to be positively related to both ToM and FI. Unexpectedly, it was negatively related to affective empathy. Moreover, according to our second hypothesis, ToM was positively related to affective empathy, but again unexpectedly, affective empathy was negatively linked to SA. Concerning the relationship of FI to SA, our results confirm the indirect relationship (hypothesis 4) of FI to SA through CI, while disproving the proposed direct relationship (hypothesis 3). In summary, FI is directly related to CI and indirectly related to SA; FI was directly related to CI and indirectly related SA. That is to say, the role of FI in predicting SA is through CI. We will discuss and further interpret these results in the following sections.

### Executive functioning, social cognition, and social adaptation

Concerning the influence of EF on SA, we identified a positive relationship between EF and ToM (a component of SC), which was moderately associated with affective empathy (statistically, it was marginally significant). However, affective empathy was negatively related to SA.

This first relationship was expected: SC (ToM) together with EF have been shown to play a crucial role in the regulation of social interaction^80,81^. Impairment in SC has also been observed among individuals who show social adaptation impairments such as those with mental health disorders^82^ and neurodegenerative diseases^83^ as well as in delinquents^84^ and ex-combatants^85,86^. Impairments in socio-affective variables—specifically, low internal locus of control, self-esteem, and high stress—have been also described among individuals living in vulnerable environments^87^.

Paradoxically, EF exhibited a strong negative correlation with affective empathy—a component of SC that was inversely related to SA in this population (although this was statistically marginally significant). This runs contrary to the findings of Sun et al. whose data suggest that poor individuals who exhibit greater affective empathy also exhibit greater coping strategies that support SA^35^. The non-replication of these results may stem from differences in the sample populations. This study focused on adults living in socially vulnerable conditions since childhood. This seems relevant given that certain areas with disproportionately low SES can be perceived as territorial traps for the most disadvantaged. In these areas, financial and professional opportunities can be scarce and resources to progress are inaccessible to most. The population recruited in this study exhibits low social mobility. Typically, several generations of their families have remained in the same place. According to an OECD report in 2019, the high levels of inequality in Chile tend to hamper income and social mobility^88^. For instance, it could take six generations for descendants of a family located in the lowest 10% of the income distribution to reach the average income compared to only four to five generations on average in all OECD countries^88^. This is also reinforced by Delaunay^89^ who states, “in Chile, the propensity to move, whether it is migration or daily mobility, increases in general with the socioeconomic level of the people, from which it follows that those of the lower strata have few options to use migration as a resource to get out of poverty” [89; p.1]. Sun et al. focused on people with low SES at the time the study was conducted with no control for maintenance or duration of SES^35^. Extended duration of impoverishment with origins in childhood may play a role in reversing the socially protective effects provided by affective empathy in populations living in poverty whose SES has sustained over their life course.

Our results align with other studies that pointed out that empathy is cognitively taxing, and thus, people tend to avoid it. Across 11 studies and a meta-analytic review, a strong preference to avoid empathy and as well as a tendency to feature conceptions of empathy as being more effortful and aversive and less efficacious than an alternative course of action have been identified^90^. We speculate that this could explain the negative relationship that we found between EF and affective empathy. Better EF abilities in adults living in poverty could assist them in better self-regulating responses to potential vicarious distress that could trigger a high empathic concern with other people. This empathy regulation—the inhibition of empathy-related behaviors and the avoidance of empathy-involved situations—to avoid distress, could, in turn, thwart SA. This theory should be addressed in future research among controlled populations of varying SES for validation.

### Executive functioning, intelligence and social adaptation

Our results showed a positive correlation between EF and FI, which was indirectly associated with SA through CI. Previous research has supported a positive reciprocal relationship between EF and FI^91^ suggesting that changes in both domains might operate bidirectionally. Among the various EFs, it is WM that has been most strongly associated with both FI and CI^92–96^. Executive functions are critical for activating, maintaining, and selecting appropriate actions or thoughts to achieve goals; they are central to self-control and self-regulation^97^ which aids mental, social, and physical long-term adaptation in “normalized” environments^98,99^. On the contrary, disadvantaged social environments promote low executive functioning (EF)^13,17,100^. Individuals that have to deal with a high amount of life stress and unpredictability may have deficits both in working memory capacity^100^—due to the added burden of chronic inhibition of unwanted thoughts about adverse life events—and in the inhibition of prepotent responses^101^.

Additionally, the finding that EF predicts FI and CI and that FI predicts SA through CI was expected as the relationship between intelligence and SA has been tested previously^10^. Recently, Huepe and Salas suggested that the elucidation of prefrontal cortex functions such as FI and perspective changing is crucial for understanding psychosocial adaptation mechanisms^102^. We did not find a direct effect of FI, but the comparison with other previous studies is not straightforward. In our model, the direct effect indicates the influence of FI on SA *controlling for* CI, whereas other studies have only tested the linear relationship between FI and SA without controlling for CI^10,87^. While it has been suggested that lower levels of education and overall socioeconomic deprivation could limit the use of FI^10^, other lines of research have suggested that intelligence itself is a predictor of poverty and health inequalities^103,104^. The combined role of this domain remains elusive; whether intelligence can be considered as a driver or an outcome of living in vulnerable conditions is unclear. Our results suggest that FI is more strongly associated with SA given its relationship to CI. These results corroborate those of a previous study^105^ that found that FI and CI could act as cognitive reserve that could have potential protective effects for people living in vulnerable contexts. Further research on the function and interaction of intelligence as it relates to SA may help to support the position of intelligence as a target for therapeutic exercise to improve psychosocial wellbeing in vulnerable populations. Systematic work in vulnerable contexts suggests that despite the limited capacity for improvement exhibited by cognitive functions such as intelligence and EF in childhood, developing therapeutic programs that favor empathy, the use of intelligence and some cognitive functions (such as self-regulation and decision-making control) allow for greater SA^106,107^.

The methods and findings described above present several limitations. First, the Social Adaptation Self-evaluation Scale (SASS) used to measure SA in participants incorporates issues related to social desirability bias into our results. Despite the above, the instrument showed good psychometric indicators and the results were consistent with what was expected (predictive validity). Notwithstanding, future research on this topic should employ SA measurement methods that encompass aspects of peer evaluation to provide comparison and reduce this bias. Second, we are aware that the WAIS-III assessment used in this study was normed in 1998. However, taking that and the date of publication of the norms of WAIS-IV for the Chilean population (2014) into account, we deemed appropriate the omission of standardized scores for IQ and rather used the direct scores of two subscales with good reliability estimates. We think that this guarantees the accuracy of the measure.

Additionally, participants recruited for this study did not include individuals of higher SES. Though conclusions were made based on the data set presented by our study, putative explanations provided by comparison to previous peer-reviewed research of populations with differing SES and living conditions require further research to substantiate. In future research, a more exhaustive and diverse sample population should be recruited to include those who have sustained high SES over their lifetime, those who lived with low SES in childhood and later lived with high SES, and those who lived with high SES in childhood and later lived with low SES. Research of this kind might yield more accurate comparisons, claims, and explanations of etiology in controlling for factors not directly isolated in this study that may impact cognition and SA.

## Conclusions

The results presented by this study support the validity and significance of EF, SC, and intelligence in predicting SA among adults living in vulnerable conditions since childhood. Specifically, the findings suggest that EF plays a fundamental role in the manifestation of SA in this population. Further longitudinal research among populations of varying SES—in both level and duration sustained—is required to validate the potential explanations suggested by the findings of this study and to inform effective intervention methods to promote psychosocial health among people living in vulnerable conditions across life stages.

## Supplementary Information

Supplementary Information.

## Data Availability

For requesting access to the full database that support the manuscript, please write to the corresponding authors.
