# Supplementary material for: The role of executive functions, social cognition and intelligence in predicting social adaptation of vulnerable populations
Source: Sci Rep. 2022 Nov 4;12:18693. doi: 10.1038/s41598-022-21985-9 (PMC9636196; doi:10.1038/s41598-022-21985-9)
Supplement: Supplementary file 1 — Supplementary Information. [file 41598_2022_21985_MOESM1_ESM.pdf]

Supplementary material 1. Alternative models.

Here, we present two alternative models that were compared to the structural equation model 1 (Model 1 from here on). Panel A in Supplementary Figures 1 and 2 shows a schematic representation of both alternative models. The aim of the implementation of these models was to examine the role of EF and FI in predicting ToM. In comparison to Model 1, structural equation model 2 (Model 2 from here on) included an additional path from FI to ToM; in the remaining paths, both models were identical. The fit of Model 2 was excellent (see Panel B in Supplementary Figure 1) and parameter estimates were very close to those exhibited in Model 1. A crucial aspect in Model 2 was the nonsignificant path from FI to ToM. These findings suggest that, in presence of an effect of EF to ToM, the influence of FI to ToM was not important. Structural equation model 3 (Model 3 from here on) resembled Model 2 (see Panel A in Supplementary Figure 2) but the former model did not include the path from EF to ToM. Again, the goodness-of-fit of Model 3 to the data was excellent (see Panel B in Supplementary Figure 2) and parameter estimates were very similar to those showed by Model 1 and Model 2. In Model 3, the path from FI to ToM was statistically significant. For model selection we used the Akaike's Information Criterion (AIC) which is based on the model log-likelihood. In this criterion, smaller values indicate the preferred model. To disambiguate the model selection process, we employed the ΔAIC between the model with the smallest value and other candidate models in the set. ΔAIC values between 0 and 2 suggest limited support to distinguish between models; values between 4 to 7 indicate less support for the model with the higher value. A difference greater than 10 indicates no support for the model with the higher value (Burnham & Anderson, 2002). Supplementary Table 1 presents the AIC and ΔAIC for all models. As the table displays, Model 1 presents the smallest AIC and ΔAIC indicates no support for Model 3. Furthermore, ΔAIC did not allow for distinguishing between Model 1 and Model 2. However, the additional parameter of Model 2 (i.e., the path from FI to ToM) was statistically nonsignificant suggesting that it is not necessary to include it in the model. Taken together, we selected Model 1 as the more parsimonious account of the data.

A)

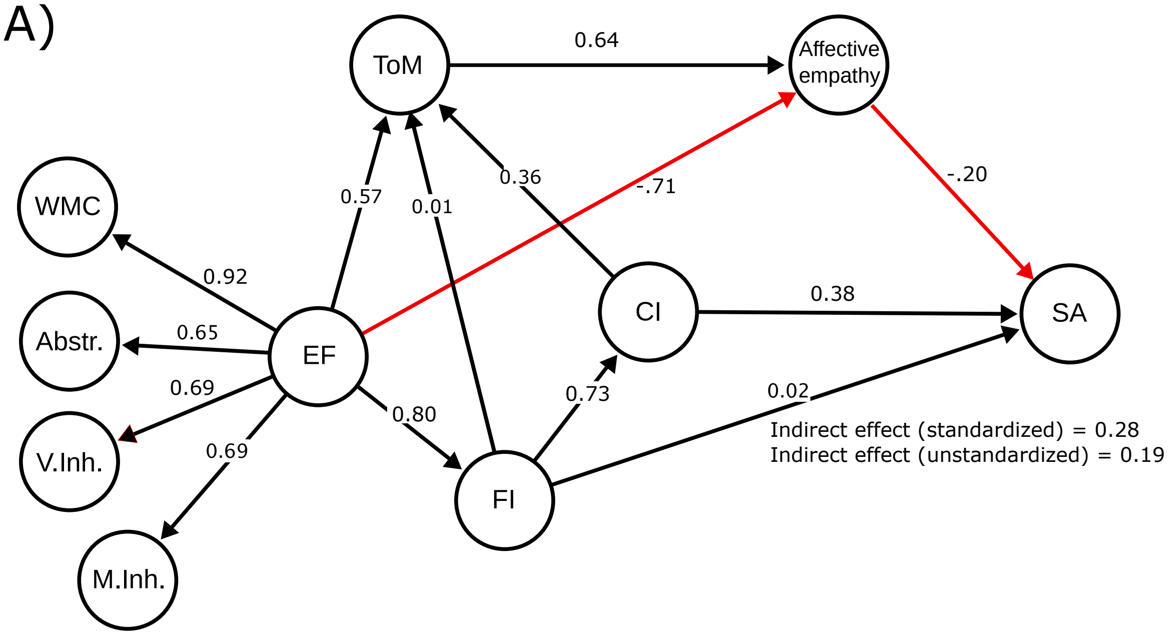

B) **Model Fit**

Yuan-Bentler X^2= 355.671

df = 335, p-value = 0.209

Robust CFI= 0.989

Robust RMSEA= 0.01[0.00 - 0.03]

Supplementary Figure 1: Structural equation modelling of social adaptation of vulnerable populations. A) Structural equation model 2; B) Model fit, values in brackets denote 90% confidence intervals. Variables in circles are unobserved (latent) factors explaining observed (manifest) variables (not shown). Arrows indicate the hypothesized pathways with numbers as the standardized regression estimates. WMC: working memory capacity; Abstr.: abstraction; V.Inh : verbal inhibitory control; M.Inh: motor inhibitory control; EF: executive function; ToM: theory of mind; FI: fluid intelligence; Affective empathy: affective empathy; SA: social adaptation; M: marginal effect.

A)

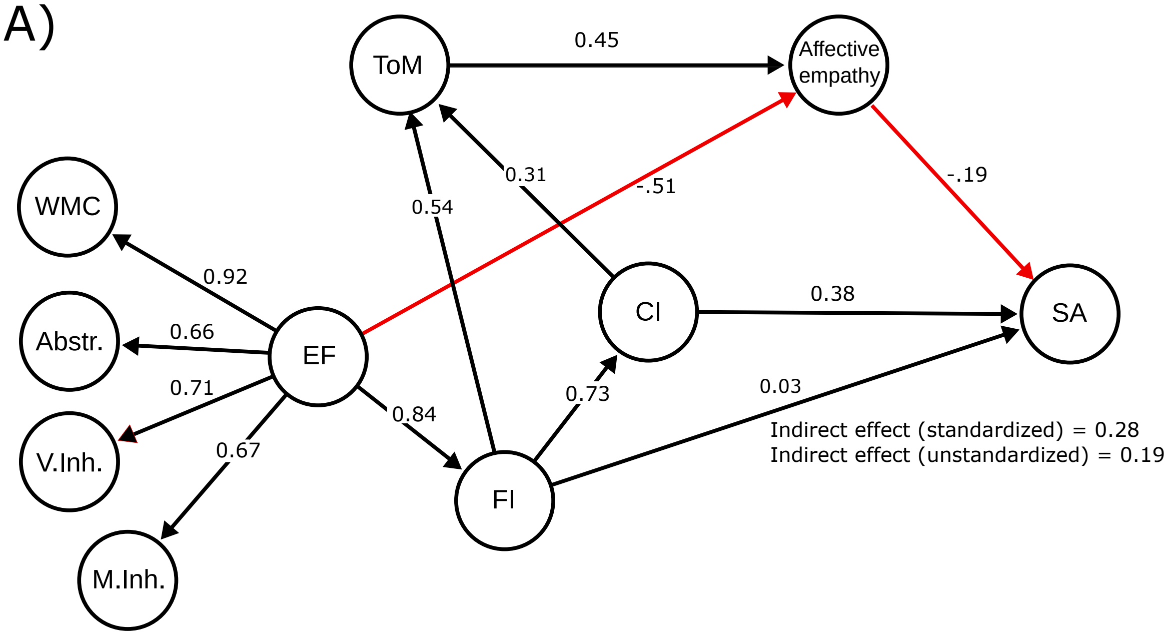

B) **Model Fit**
Yuan-Bentler X^2= 367.789
df = 336, p-value = 0.112
Robust CFI= 0.98
Robust RMSEA= 0.02[0.00 - 0.03]

Supplementary Figure 2: Structural equation modelling of social adaptation of vulnerable populations. A) Structural equation model 3; B) Model fit, values in brackets denote 90% confidence intervals. Variables in circles are unobserved (latent) factors explaining observed (manifest) variables (not shown). Arrows indicate the hypothesized pathways with numbers as the standardized regression estimates. Dashed lines indicate that the effects were not statistically significant. WMC: working memory capacity; Abstr.: abstraction; V.Inh : verbal inhibitory control; M.Inh: motor inhibitory control; EF: executive function; ToM: theory of mind; FI: fluid intelligence; Affective empathy: affective empathy; SA: social adaptation; M: marginal effect.

Supplementary Table 1. Goodness-of-fit indexes for model selection.

Model	*df*	AIC	ΔAIC
**Structural equation model 1**	336	5449.8	0.0000
**Structural equation model 2**	335	5451.8	1.9984
**Structural equation model 3**	336	5463.0	13.1562

Note. *df*: degree of freedom; AIC: Akaike's Information Criterion; ΔAIC: AIC differences.

References

Burnham, K. P., & Anderson, D. R. (2002). *Model Selection and Multimodel Inference: A Practical Information-Theoretic Approach*. Springer-Verlag. http://sutlib2.sut.ac.th/sut_contents/H79182.pdf
